# A System of Human Anatomy, General and Special

**Published:** 1848

**Authors:** 


### ARTICLE II.

*A System of Human Anatomy, General and Special.* By ERASMUS WILSON, M. D., Lecturer on Anatomy, London. Fourth American, from the last London edition. Edited by PAUL B. GODDARD, A. M., M. D., Professor of Anatomy and Histology in the Franklin Medical College of Philadelphia. Philadelphia: Lea & Blanchard. 1848. pp. 376. (From the publishers: for sale by Keen & Bro., Chicago.)

The fact that this most excellent work has been so generally adopted as the text-book of Anatomy in this country as to require and justify the publication of four editions in as many years, is sufficient evidence of its popularity. We have always, since its first appearance, recommended it as affording the most concise, clear, and minute descriptions of any book on Anatomy. Since the publication of the first edition, as each has appeared in quick succession, additions and improvements have been made, some by the author, and others no less valuable by the editor; so that it may now be said to be equal to any, if not the very best, work upon this important branch of medical science. H.

